# Supplementary material for: ABHD2 activity is not required for the non-genomic action of progesterone on human sperm
Source: Hum Reprod. 2026 May 29;41(8):1409–19. doi: 10.1093/humrep/deag085 (PMC13429874; doi:10.1093/humrep/deag085)

Supplement

Supplementary materials and methods

DNA sequences

ABHD2-c051 coding sequence

ATGCATCATCACCATCACCATGACTACAAGGACGATGACGACAAGGAGAACCTTTATTT
TCAAGGATTGAACCTGAAGAGCCCCACAGCCCCACCTGACCTCTACTTCCAGGACTCG
GGGCTCTCACGCTTTCTGCTCAAGTCCTGTCCTCTTCTGACCAAAGAATACATTCCACC
GTTGATCTGGGGGAAAAGTGGACACATCCAGACAGCCTTGTATGGGAAGATGGGAAGG
GTGAGGTCGCCACATCCTTATGGGCACCGGAAGTTCATCACTATGTCTGATGGAGCCA
CTTCTACATTCGACCTCTTCGAGCCCTTGGCTGAGCACTGTGTTGGAGATGATATCACC
ATGGTCATCTGCCCTGGAATTGCCAATCACAGCGAGAAGCAATACATCCGCACTTTCGT
TGACTACGCCCAGAAAAATGGCTATCGGTGCGCCGTGCTGAACCACCTGGGTGCCCTG
CCCAACATTGAATTGACCTCGCCACGCATGTTCACCTATGGCTGCACGTGGGAATTTGG
AGCCATGGTGAACTACATCAAGAAGACATATCCCCTGACCCAGCTGGTCGTCGTGGGC
TTCAGCCTGGGTGGTAACATTGTGTGCAAATACTTGGGGGAGACTCAGGCAAACCAAG
AGAAGGTCCTGTGCTGCGTCAGCGTGTGCCAGGGGTACAGTGCACTGAGGGCCCAGG
AAACCTTCATGCAATGGGATCAGTGCCGGCGGTTCTACAACTTCCTCATGGCTGACAAC
ATGAAGAAGATCATCCTCTCGCACAGGCAAGCTCTTTTTGGAGACCATGTTAAGAAACC
CCAGAGCCTGGAAGACACGGACTTGAGCCGGCTCTACACAGCAACATCCCTGATGCAG
ATTGATGACAATGTGATGAGGAAGTTTCACGGCTATAACTCCCTGAAGGAATACTATGA
GGAAGAAAGTTGCATGCGGTACCTGCACAGGATTTATGTTCCTCTCATGCTGGTTAATG
CAGCTGACGATCCGTTGGTGCATGAAAGTCTTCTAACCATTCCAAAATCTCTTTCAGAG
AAACGAGAGAACGTCATGTTTGTGCTGCCTCTGCATGGGGGCCACTTGGGCTTCTTTG
AGGGCTCTGTGCTGTTCCCCGAGCCCCTGACATGGATGGATAAGCTGGTGGTGGAGTA
CGCCAACGCCATTTGCCAATGGGAGCGTAACAAGTTGCAGTGCTCTGACACGGAGCAG
GTGGAGGCCGACCTGGAGTGA

ABHD2-c054 coding sequence

ATGCATCATCACCATCACCATGACTACAAGGACGATGACGACAAGGAGAACCTTTATTT
TCAGGGCTTGAACCTGAAGAGCCCCACAGCCCCACCTGACCTCTACTTCCAGGACTCG
GGGCTCTCACGCTTTCTGCTCAAGTCCTGTCCTCTTCTGACCAAAGAATACATTCCACC
GTTGATCTGGGGGAAAAGTGGACACATCCAGACAGCCTTGTATGGGAAGATGGGAAGG
GTGAGGTCGCCACATCCTTATGGGCACCGGAAGTTCATCACTATGTCTGATGGAGCCA
CTTCTACATTCGACCTCTTCGAGCCCTTGGCTGAGCACTGTGTTGGAGATGATATCACC

ATGGTCATCTGCCCTGGAATTGCCAATCACAGCGAGAAGCAATACATCCGCACTTTCGT
TGACTACGCCCAGAAAAATGGCTATCGGTGCGCCGTGCTGAACCACCTGGGTGCCCTG
CCCAACATTGAATTGACCTCGCCACGCATGTTCACCTATGGCTGCACGTGGGAATTTGG
AGCCATGGTGAACTACATCAAGAAGACATATCCCCTGACCCAGCTGGTCGTCGTGGGC
TTCGCGCTGGGTGGTAACATTGTGTGCAAATACTTGGGGGAGACTCAGGCAAACCAAG
AGAAGGTCCTGTGCTGCGTCAGCGTGTGCCAGGGGTACAGTGCACTGAGGGCCCAGG
AAACCTTCATGCAATGGGATCAGTGCCGGCGGTTCTACAACTTCCTCATGGCTGACAAC
ATGAAGAAGATCATCCTCTCGCACAGGCAAGCTCTTTTTGGAGACCATGTTAAGAAACC
CCAGAGCCTGGAAGACACGGACTTGAGCCGGCTCTACACAGCAACATCCCTGATGCAG
ATTGATGACAATGTGATGAGGAAGTTTCACGGCTATAACTCCCTGAAGGAATACTATGA
GGAAGAAAGTTGCATGCGGTACCTGCACAGGATTTATGTTCCTCTCATGCTGGTTAATG
CAGCTGACGATCCGTTGGTGCATGAAAGTCTTCTAACCATTCCAAAATCTCTTTCAGAG
AAACGAGAGAACGTCATGTTTGTGCTGCCTCTGCATGGGGGCCACTTGGGCTTCTTTG
AGGGCTCTGTGCTGTTCCCCGAGCCCCTGACATGGATGGATAAGCTGGTGGTGGAGTA
CGCCAACGCCATTTGCCAATGGGAGCGTAACAAGTTGCAGTGCTCTGACACGGAGCAG
GTGGAGGCCGACCTGGAGTGA

Chemical synthesis

All chemical reagents were purchased from Sigma-Aldrich, Merck, Fisher Scientific, Apollo Scientific, or Fluorochem and used without further purification. Solvents were evaporated under reduced pressure using a Büchi rotary evaporator. Room temperature is defined as between 18–22 °C. Thin-layer chromatography (TLC) was performed on Merck Silica Gel 60 F 254 precoated aluminium plates (0.2 mm) visualised with UV irradiation (254 nm) and stained with aqueous basic potassium permanganate solution. Reverse phase flash column chromatography was carried out using Biotage® Sfär C18 columns and the Biotage® Selekt flash purification system. 1H and 13C NMR spectra were recorded using Bruker Avance III 400. Chemical shifts (δ) are quoted in parts per million (ppm) and are referenced to the residual solvent peak: CDCl3 (δ 7.26 ppm in 1H and δ 77.2 ppm in 13C). Coupling constants (J) are quoted in Hertz (Hz). The 1H NMR spectra are reported as follows: ppm (multiplicity, coupling constants, number of protons, assignment). NMR assignments use numbering independent from IUPAC, using two-dimensional (COSY, HSQC, HMBC) NMR spectroscopy to assist the assignment. Analytical liquid chromatography mass spectrometry (LCMS) was performed on an Agilent Infinity 1260 II system consisting of a quaternary pump (G7111A) and a diode array detector WR (G7115A) coupled to an InfinityLab LC/ MSD (G6125B) using ESI. An Agilent Poroshell 120 EC-C18 column (2.7 µm, 4.6 mm × 50 mm) was used at a flow rate of 1.5 mL/min with a mobile phase of 0.05% formic acid in H2O and 0.05% formic acid in MeCN.

High-resolution mass spectrometry (HRMS) was performed on an Agilent 6545XT AdvanceBio LC/Q-TOF using ESI in positive detection. Chiral high-performance liquid chromatography (HPLC) was performed on an Agilent Infinity 1200 system consisting of a quaternary pump (G1311A) and a diode array detector (G1315D) using a Chiralcel OD-H column.

General Procedure A:

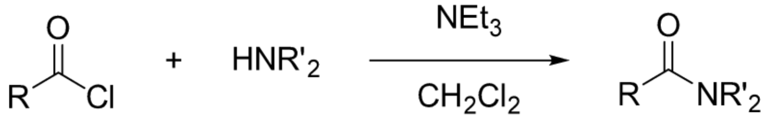

To amine (0.20 mmol, 1.0 equiv.) in CH_2Cl_2 (1.0 mL) under argon was added triethylamine (0.60 mmol, 3.0 equiv.) then acyl chloride (0.30 mmol, 1.5 equiv.). The reaction was stirred at room temperature and monitored by LCMS. If, after 2 h, a significant quantity of amine starting material was present, more triethylamine (0.60 mmol, 3.0 equiv.) then acyl chloride (0.30 mmol, 1.5 equiv.) were added. The sample was concentrated under reduced pressure to provide crude product. Reverse phase flash column chromatography was performed (5–100% methanol in water) with UV detection at 208 nm to give the product.

General Procedure B:

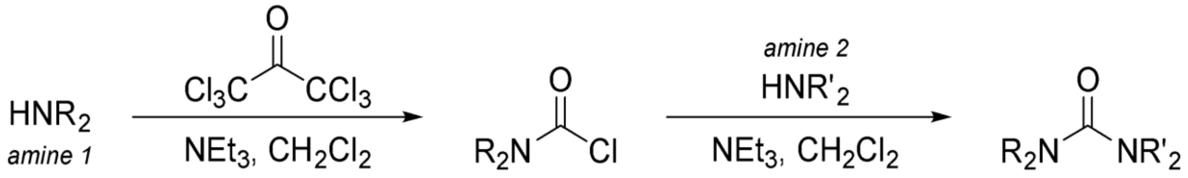

To amine 1 (0.20 mmol, 1.0 equiv.) in CH_2Cl_2 (1.0 mL) under argon at 0 °C was added triethylamine (0.40 mmol, 2.0 equiv.) then triphosgene (0.20 mmol, 1.0 equiv.). The reaction was stirred at 0 °C until completion as indicated by TLC. In another flask, to amine 2 (0.60 mmol, 3.0 equiv.) in CH_2Cl_2 (1.0 mL) under argon at room temperature was added triethylamine (0.60 mmol, 3.0 equiv.). This was added to the previous solution dropwise at 0 °C then stirred at room temperature and monitored by LCMS. The sample was concentrated under reduced pressure to provide crude product. Reverse phase flash column chromatography was performed (5–100% methanol in water) with UV detection at 208 nm to give the product.

(2-Benzylpiperidin-1-yl)(piperidin-1-yl)methanone (Compound **1**)

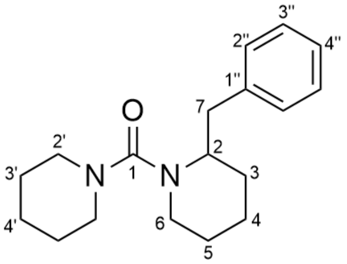

Prepared using general procedure A in 64% yield for racemic, 72% for (*R*)-isomer and 58% for (*S*)-isomer as clear oils. **^{1}H NMR** (400 MHz, $CDCl_3$) δ 7.29 – 7.23 (m, 2H, 3''-H), 7.22 – 7.14 (m, 3H, 2''-H and 4''-H), 4.22 – 4.03 (m, 1H, 2-H), 3.50 – 3.35 (m, 1H, one of 6-H), 3.14 – 2.70 (m, 7H, 2'-H, 7-H and one of 6-H), 1.80 – 1.32 (m, 12H, 3'-H, 4'-H, 3-H, 4-H and 5-H); **^{13}C NMR** (101 MHz, $CDCl_3$) δ 165.1 (C-1), 139.7 (C-1''), 129.3 (C-2''), 128.5 (C-3''), 126.2 (C-4''), 54.8 (C-2), 48.2 (C-2'), 42.9 (C-6), 36.2 (C-7), 27.2 (C-3), 25.9 (C-5), 25.9 (C-3'), 24.9 (C-4'), 19.6 (C-4); ***m/z*** [ESI+] 287 ($[M+H]^+$, 100%), 595 ($[2M+Na]^+$, 48%); **Chiral HPLC** was performed using an OD-H column and UV detection at 208 nm using an isocratic mobile phase of 2:98 isopropanol:*n*-hexane over 20 min at 1 mL/min. The racemic **1** eluted at a retention time of 8.9 and 10.3 min. *(R)*-**1** (*R*-isomer) eluted at 9.2 min and *(S)*-**1** (*S*-isomer) eluted at 10.3 min, each as single enantiomers. The spectroscopic data match those in the literature.[33]

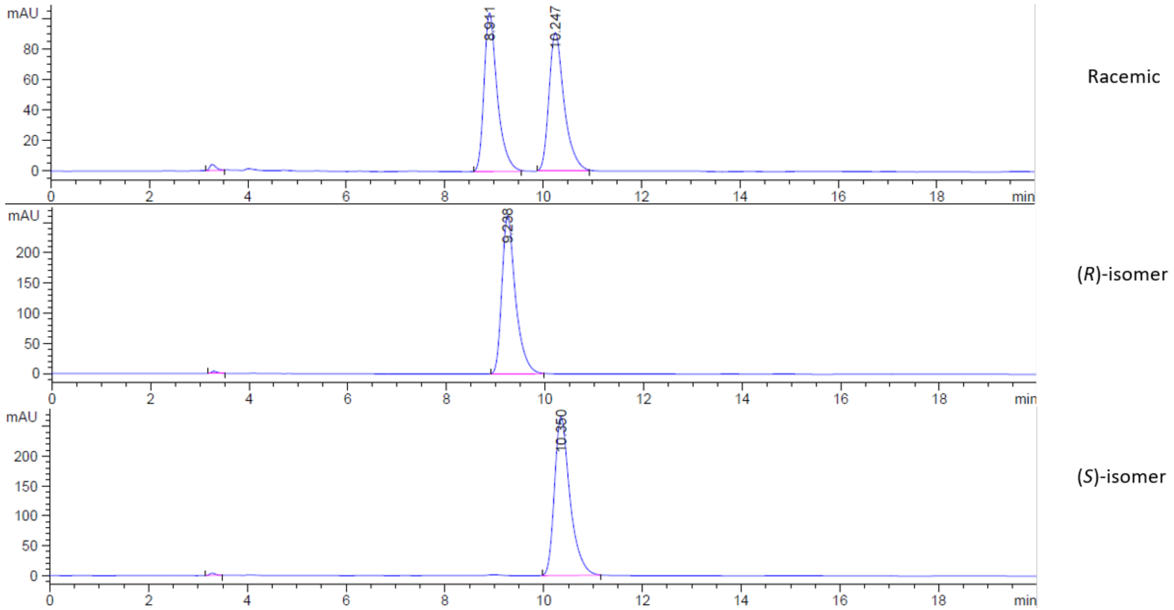

(2-Phenylpiperidin-1-yl)(piperidin-1-yl)methanone (Compound **2**)

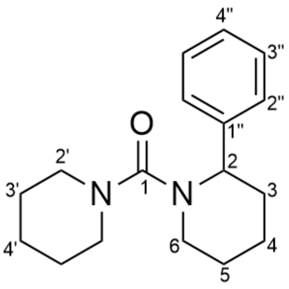

Prepared using general procedure A in 88% yield for racemic, 91% for (*R*)-isomer and 90% for (*S*)-isomer as clear oils. **^{1}H NMR** (400 MHz, $CDCl_3$) δ 7.29 – 7.15 (m, 4H, 2''-H and 3''-H), 7.13 (m, 1H, 4''-H), 4.75 (t, *J* = 5.0 Hz, 1H, 2-H), 3.30 (m, 1H, one of 6-H), 3.19 (m, 4H, 2'-H), 2.99 – 2.88 (m, 1H, one of 6-H), 2.00 (m, 1H, one of 3-H), 1.85 (m, 1H, one of 3-H), 1.49 (m, 10H, 4-H, 5-H, 3'-H and 4'-H); **^{13}C NMR** (101 MHz, $CDCl_3$) δ 165.2 (C-1), 142.2 (C-1''), 128.6 (C-3''), 126.8 (C-2''), 126.5 (C-4''), 57.2 (C-2), 47.7 (C-2'), 45.6 (C-6), 30.3 (C-3), 26.0 (C-3'), 25.9 (C-

5), 24.9 (C-4'), 21.3 (C-4); ***m/z*** [ESI+] 273 ($[M+H]^+$, 100%), 295 ($[M+Na]^+$, 29%); **HRMS** [ESI+] found $[M+H]^+$ 273.1965. $C_{17}H_{25}N_2O^+$ requires 273.1961; **Chiral HPLC** was performed using an OD-H column and UV detection at 208 nm using an isocratic mobile phase of 2:98 isopropanol:*n*-hexane over 20 min at 1 mL/min. The racemic **2** eluted at a retention time of 9.1 and 11.6 min. *(R)*-**2** (*S*-isomer) eluted at 9.1 min and *(S)*-**2** (*R*-isomer) eluted at 11.6 min, each as single enantiomers.

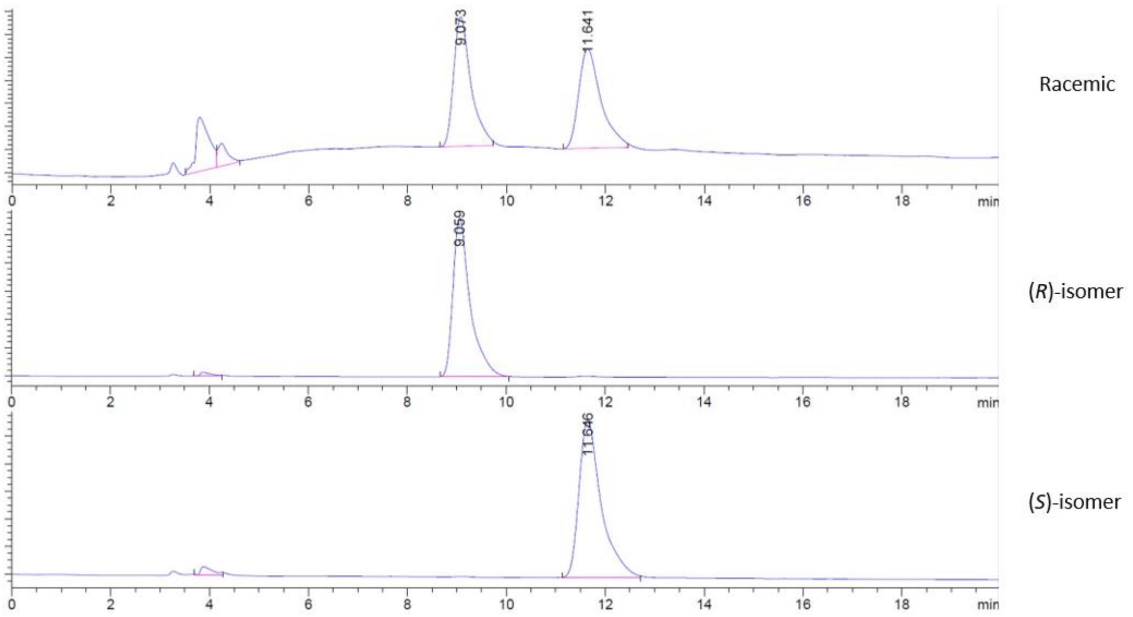

Piperidin-1-yl(2-(p-tolyl)piperidin-1-yl)methanone (Compound **5**)

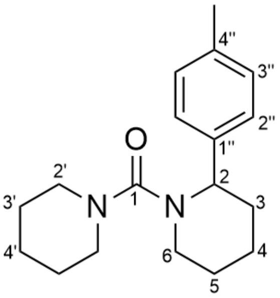

Prepared using general procedure A in 69% yield as a clear oil.

^{1}H NMR (400 MHz, $CDCl_3$) δ 7.21 – 7.10 (m, 4H, 2''-H and 3''-H), 4.79 (t, *J* = 4.8 Hz, 1H, 2-H), 3.43 – 3.33 (m, 1H, one of 6-H), 3.33 – 3.18 (m, 4H, 2'-H), 3.05 – 2.94 (m, 1H, one of 6-H), 2.32 (s, 3H, Me), 2.11 – 2.09 (m, 1H, one of 3-H), 1.96 – 1.84 (m, 1H, one of 3-H), 1.68 – 1.48 (m, 10H, 4-H, 5-H, 3'-H and 4'-H); **^{13}C NMR** (101 MHz, $CDCl_3$) δ 165.2 (C-1), 138.9 (C-1''), 136.0 (C-4''), 129.4 (C-3''), 126.7 (C-2''), 57.0 (C-2), 47.7 (C-2'), 45.4 (C-6), 30.3 (C-3), 26.0 (C-3'), 25.9 (C-5), 24.9 (C-4'), 21.2 (C-4), 21.1 (Me); ***m/z*** [ESI+] 287 ($[M+H]^+$, 100%), 595 ($[2M+Na]^+$, 74%); **HRMS** [ESI+] found $[M+H]^+$ 287.2124. $C_{18}H_{27}N_2O^+$ requires 287.2118.

(2-Benzylpiperidin-1-yl)(4-methylpiperazin-1-yl)methanone (Compound **6**)

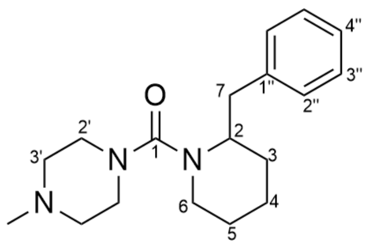

Prepared using general procedure A in 66% yield as a clear oil.

^{1}H NMR (400 MHz, $CDCl_3$) δ 7.31 – 7.23 (m, 2H, 3''-H), 7.23 – 7.13 (m, 3H, 2''-H and 4''-H), 4.32 – 4.07 (m, 1H, 2-H), 3.47 (dt, *J* = 13.3, 3.6 Hz, 1H, one of 6-H), 3.17 – 3.04 (m, 3H, one of 6-H and two of 2'-H), 3.03 – 2.91 (m, 3H, two of 2'-H and one of 7-H), 2.83 (dd, *J* = 13.5, 7.8 Hz, 1H, one of 7-H), 2.35 – 2.15 (m, 7H, 3'-H and Me), 1.85 – 1.35 (m, 6H, 3-H, 4-H and 5-H); **^{13}C NMR** (101 MHz, $CDCl_3$) δ 164.4 (C-1), 139.5 (C-1''), 129.3 (C-2''), 128.5 (C-3''), 126.3 (C-4''), 54.9 (C-3'), 54.5 (C-2), 46.9 (C-2'), 46.3 (Me), 42.9 (C-6), 36.3 (C-7), 27.4 (C-3), 25.9 (C-5), 19.5 (C-4); ***m/z*** [ESI+] 302 ($[M+H]^+$, 100%); **HRMS** [ESI+] found $[M+H]^+$ 302.2239. $C_{18}H_{28}N_3O^+$ requires 302.2227.

(2-Benzylpiperidin-1-yl)(piperidin-1-yl)methanone (Compound **1**)

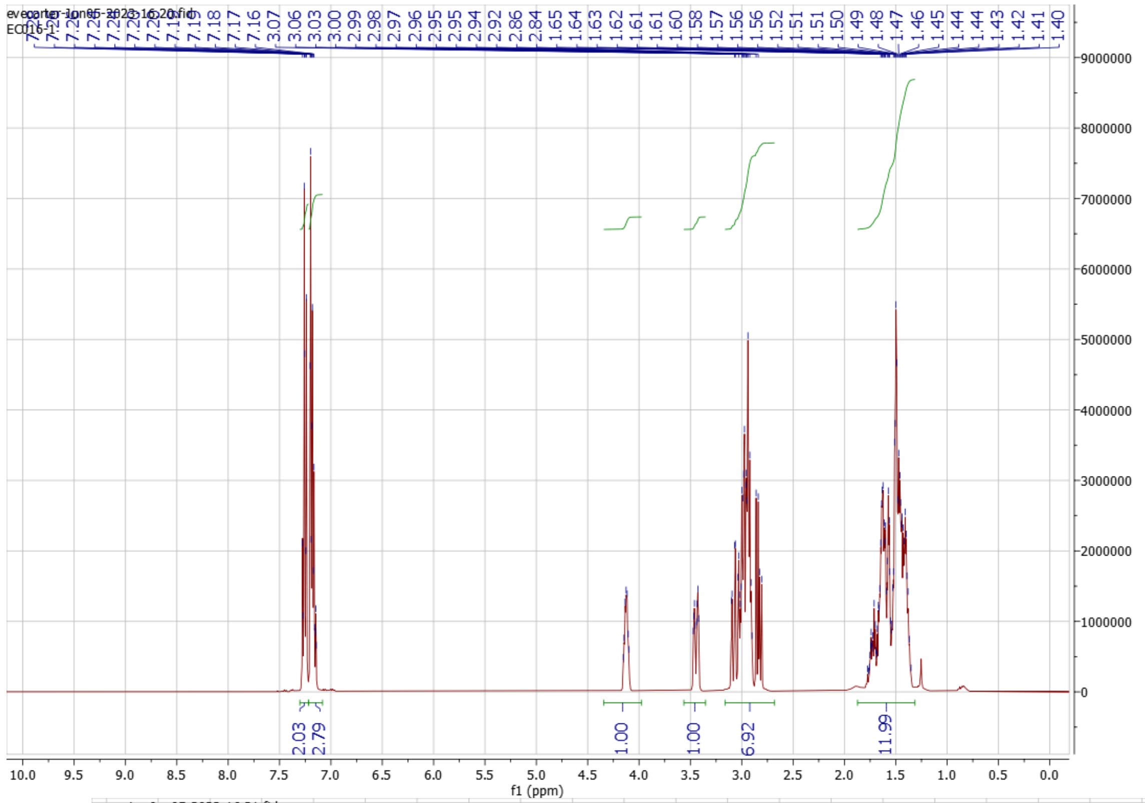

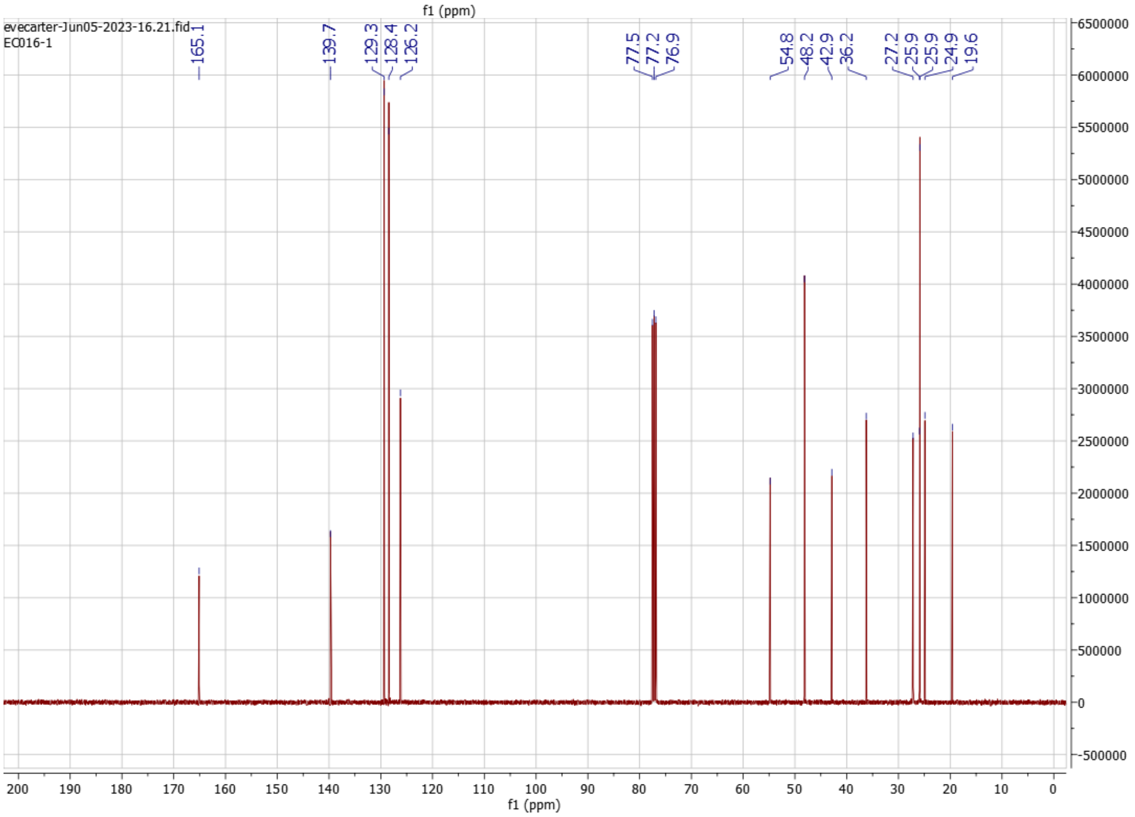

(2-Phenylpiperidin-1-yl)(piperidin-1-yl)methanone (Compound **2**)

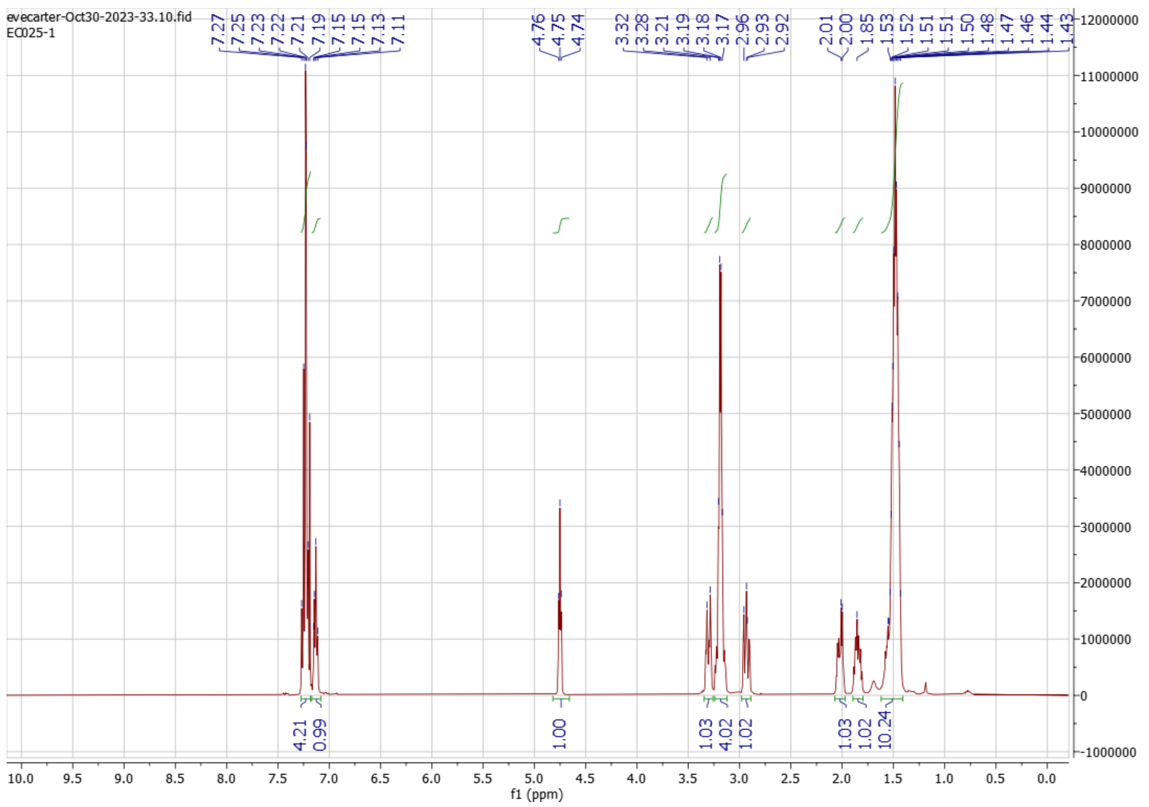

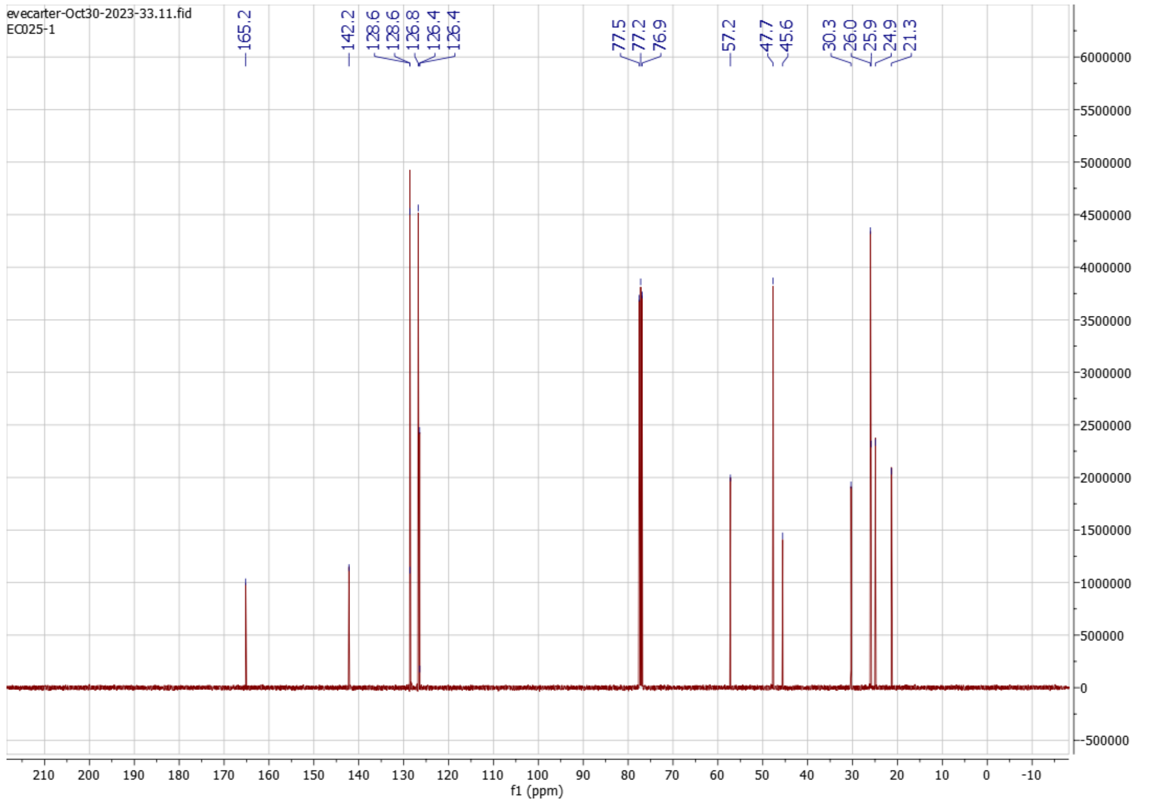

Piperidin-1-yl(2-(p-tolyl)piperidin-1-yl)methanone (Compound **5**)

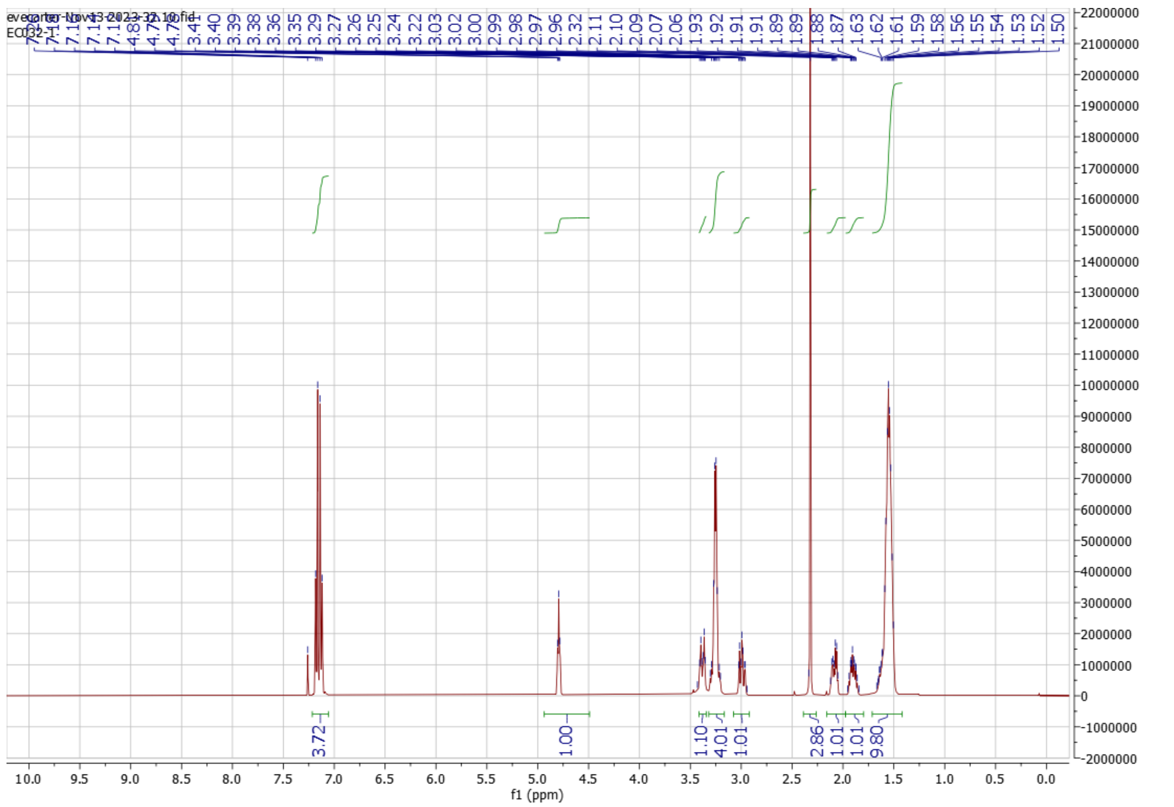

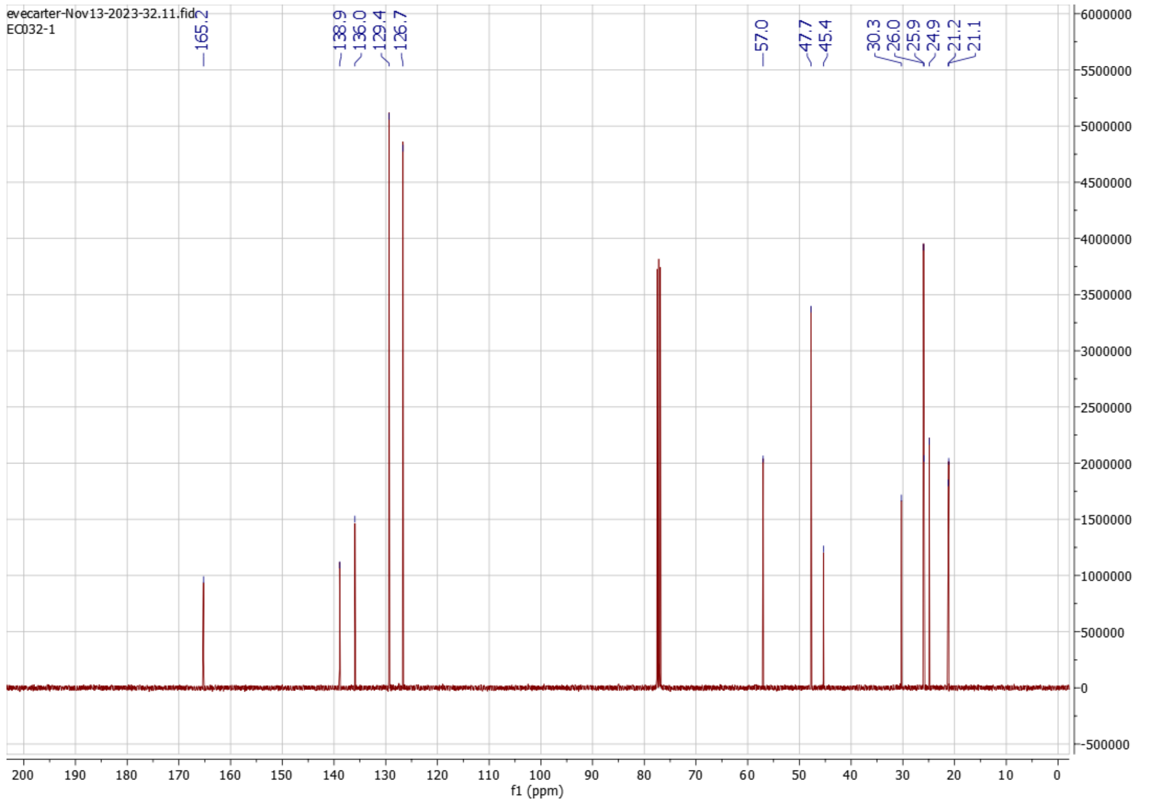

(2-Benzylpiperidin-1-yl)(4-methylpiperazin-1-yl)methanone (Compound **6**)

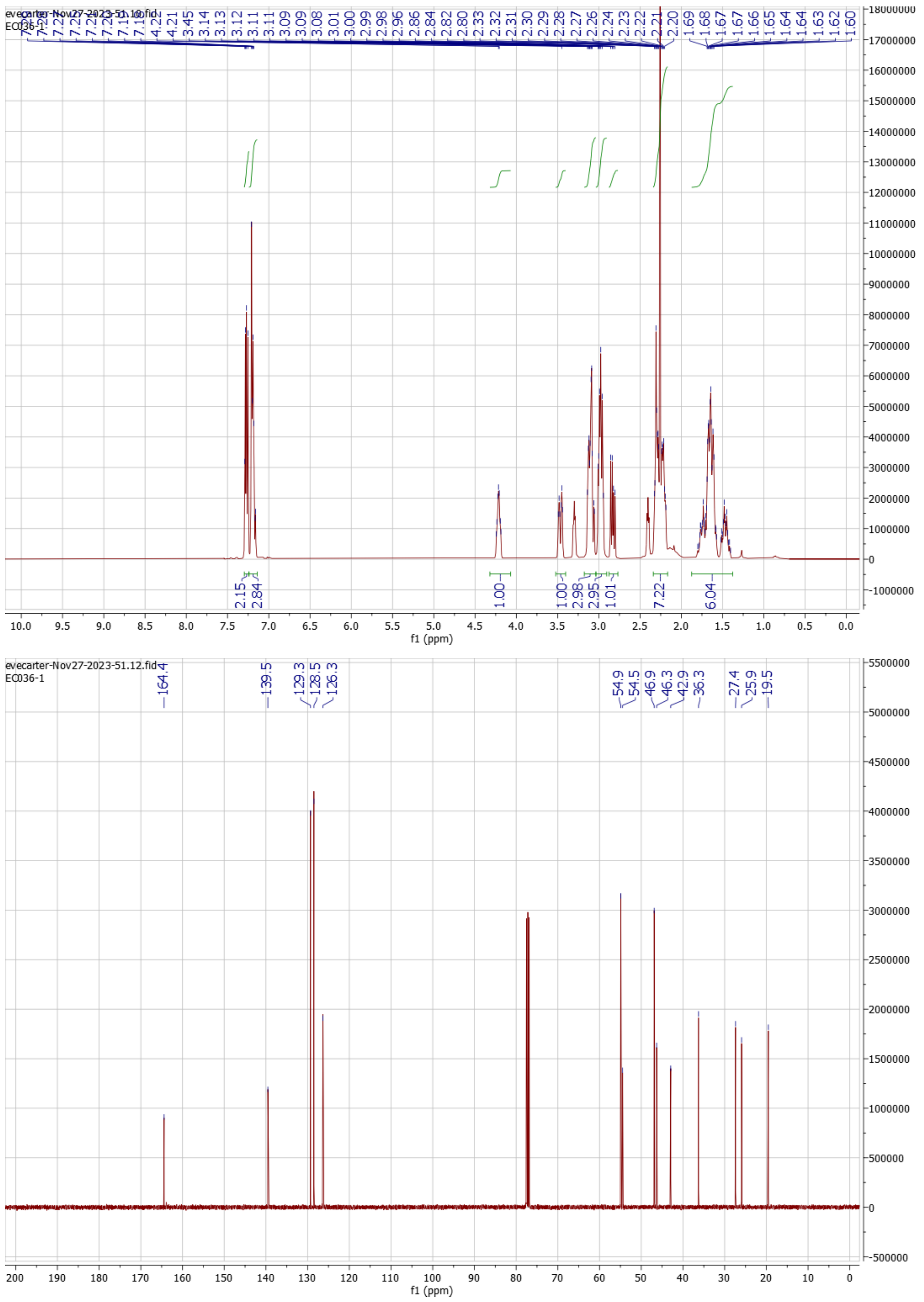

Supplement: deag085_Supplementary_materials_and_methods [file deag085_supplementary_materials_and_methods.pdf]
