# Supplementary material for: ABHD2 activity is not required for the non-genomic action of progesterone on human sperm
Source: Hum Reprod. 2026 May 29;41(8):1409–19. doi: 10.1093/humrep/deag085 (PMC13429874; doi:10.1093/humrep/deag085)
Supplement: deag085_Supplementary_Figure_S1 [file deag085_supplementary_figure_s1.pdf]

Human Reproduction, 2026, 1–1
https://doi.org/10.1093/humrep/deag085
Supplementary Data

human reproduction
OXFORD

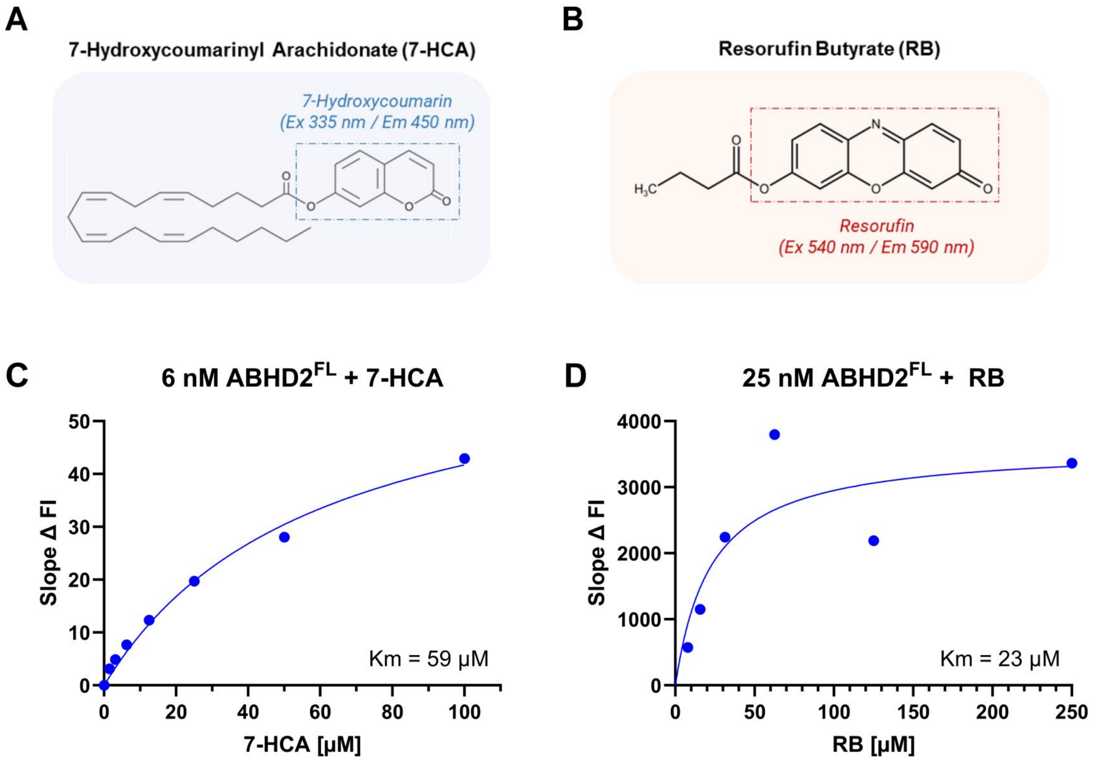

Supplementary Figure S1. Full length ABHD2 ($ABHD2^{FL}$) hydrolyzes the fluorogenic substrates 7-hydroxycoumarinyl arachidonate (7-HCA) and resorufin butyrate (RB). (**A**) Molecular structure of 7-hydroxycoumarinyl arachidonate (7-HCA). (**B**) Molecular structure of resorufin butyrate (RB). (**C**) The indicated 7-HCA concentrations were incubated with 6 nM $ABHD2^{FL}$ in assay buffer for 60 min at RT. Fluorescence was monitored every 30 s at excitation and emission wavelengths of 335 nm and 450 nm, respectively. Michaelis constant (K_m) was determined by nonlinear regression to the Michaelis–Mention equation. (**D**) The indicated RB concentrations were incubated with 25 nM $ABHD2^{FL}$ in assay buffer for 60 min at RT. Fluorescence was monitored every 30 s at excitation and emission wavelengths of 540 and 590 nm, respectively. Michaelis constant (K_m) was determined by nonlinear regression to the Michaelis–Mention equation.
