# Supplementary material for: ABHD2 activity is not required for the non-genomic action of progesterone on human sperm
Source: Hum Reprod. 2026 May 29;41(8):1409–19. doi: 10.1093/humrep/deag085 (PMC13429874; doi:10.1093/humrep/deag085)
Supplement: deag085_Supplementary_Figure_S2 [file deag085_supplementary_figure_s2.pdf]

Human Reproduction, 2026, 1–1
https://doi.org/10.1093/humrep/deag085
Supplementary Data
human reproduction
OXFORD

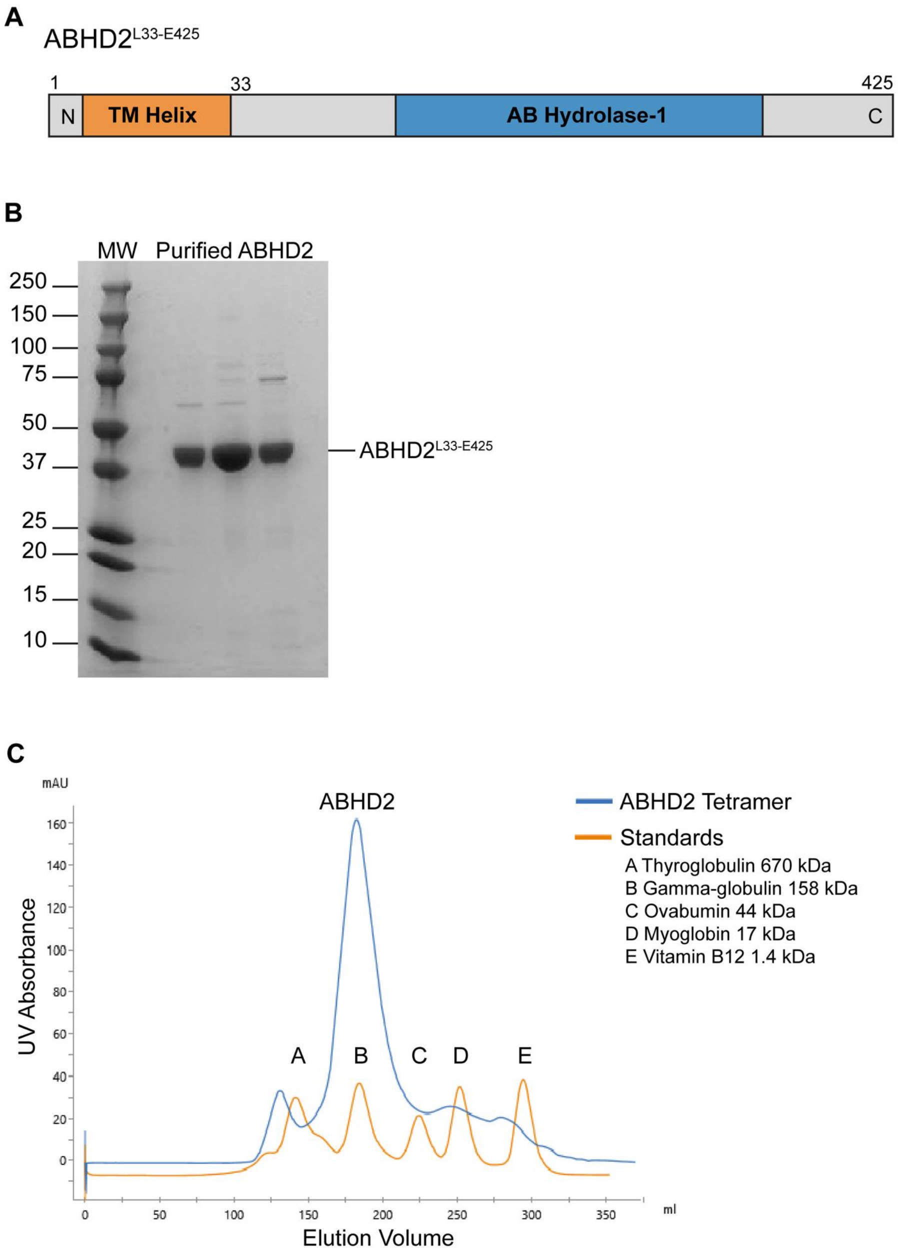

Supplementary Figure S2. Truncated ABHD2 (ABHD2$^{L33\text{-}E425}$) purification. (**A**) Domain structure of human ABHD2. For the recombinant protein, the putative transmembrane (TM) helix (amino acids 1–32) was removed. (**B**) SDS-PAGE analysis of purified ABHD2$^{L33\text{-}E425}$ after size exclusion chromatography (SEC) shows the 45 kDa protein. (**C**) SEC trace of absorbance at 280 nm (mAU) vs. elution volume (ml) from the final purification step of recombinant ABHD2$^{L33\text{-}E425}$ (blue). The SEC trace for ABHD2$^{L33\text{-}E425}$ is overlaid with the SEC trace for BioRad Chromotography Standards (orange), which were run on the same column in a separate experiment. Comparing the elution volume of ABHD2$^{L33\text{-}E425}$ to the standards allows for a predicted molecular weight that most closely corresponds to a tetramer.
