# Supplementary material for: ABHD2 activity is not required for the non-genomic action of progesterone on human sperm
Source: Hum Reprod. 2026 May 29;41(8):1409–19. doi: 10.1093/humrep/deag085 (PMC13429874; doi:10.1093/humrep/deag085)
Supplement: deag085_Supplementary_Figure_S3 [file deag085_supplementary_figure_s3.pdf]

Human Reproduction, 2026, 1–1
https://doi.org/10.1093/humrep/deag085
Supplementary Data

human reproduction

OXFORD

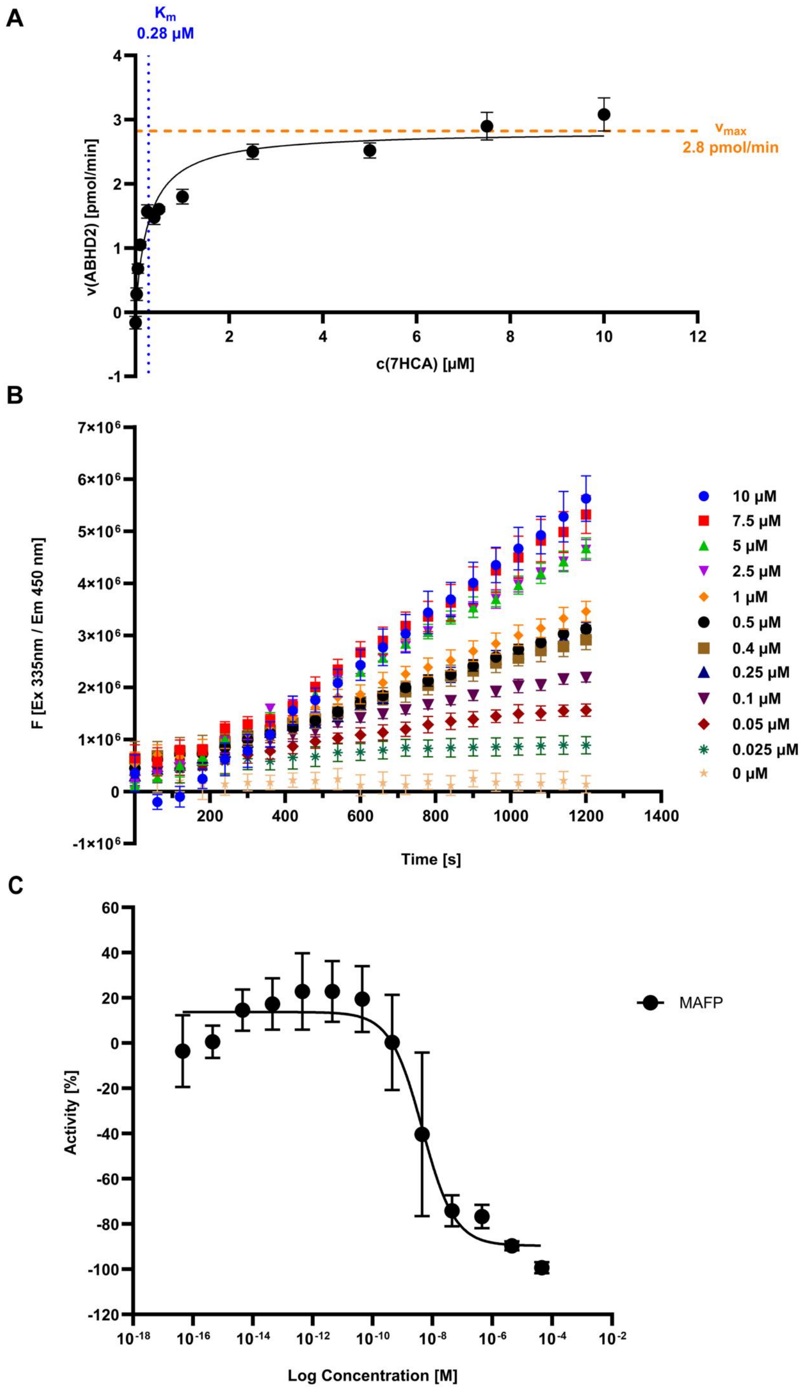

Supplementary Figure S3. Hydrolase activity of ABHD2$^{L33-E425}$. (**A**) The hydrolase activity of ABHD2$^{L33-E425}$ was assessed by a fluorometric assay using 7-hydroxycoumarinyl arachidonate (7-HCA) as substrate. A K_m and k_{cat} of 280 nM and 2.8 pmol substrate/min/pmol enzyme, respectively, were calculated, from (**B**) the original K_m curves. (**C**) ABHD2$^{L33-E425}$ is inhibited by MAFP with an IC50 of 4.6 nM.
