# Supplementary material for: ABHD2 activity is not required for the non-genomic action of progesterone on human sperm
Source: Hum Reprod. 2026 May 29;41(8):1409–19. doi: 10.1093/humrep/deag085 (PMC13429874; doi:10.1093/humrep/deag085)
Supplement: deag085_Supplementary_Figure_S4 [file deag085_supplementary_figure_s4.pdf]

Human Reproduction, 2026, 1–1
https://doi.org/10.1093/humrep/deag085
Supplementary Data

human reproduction

OXFORD

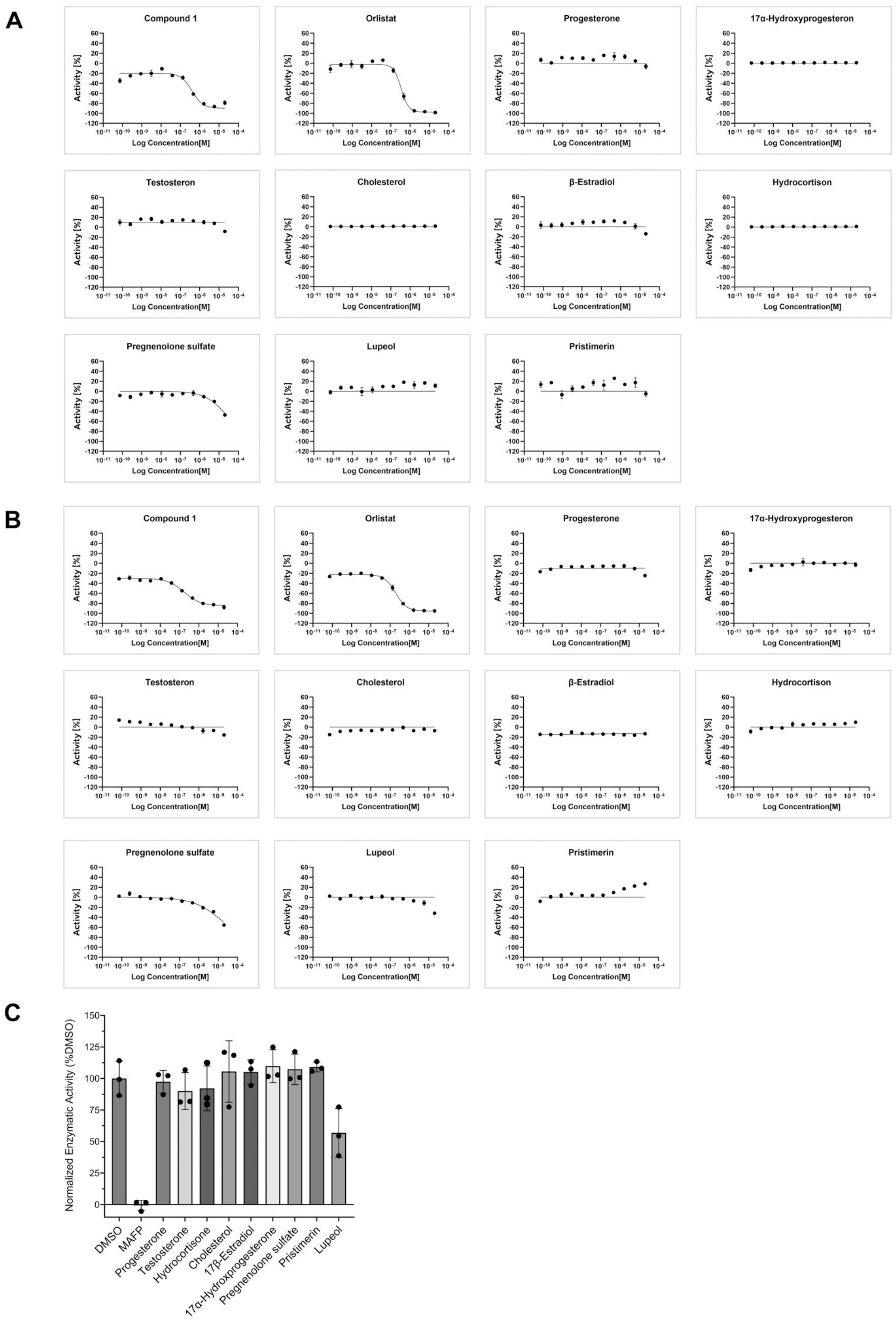

Supplementary Figure S4. Steroid hormones and triterpenoids do not modulate the enzymatic activity of ABHD2. (**A, B**) Dose–response curves were generated by incubating a fixed ABHD2^{FL} concentration with 5 μM 7-HCA (A) or 15 μM RB (B) substrate in the presence of increasing concentrations of positive controls, Compound **1** and Orlistat, the indicated steroid hormones or triterpenoids. Percent activity was plotted against log10 inhibitor concentration. (**C**) Percent activity of 10 nM ABHD2$^{L33–E425}$ in the presence of 5 μM steroids, triterpenoids, or MAFP compared to DMSO control. Percent activity is a comparison of the rate of substrate production by ABHD2$^{L33–E425}$ in the presence of steroids or triterpenoids to the rate in the presence of DMSO, across three biological replicates.
