# Supplementary material for: ABHD2 activity is not required for the non-genomic action of progesterone on human sperm
Source: Hum Reprod. 2026 May 29;41(8):1409–19. doi: 10.1093/humrep/deag085 (PMC13429874; doi:10.1093/humrep/deag085)
Supplement: deag085_Supplementary_Figure_S5 [file deag085_supplementary_figure_s5.pdf]

Human Reproduction, 2026, 1–1
https://doi.org/10.1093/humrep/deag085
Supplementary Data

human reproduction
OXFORD

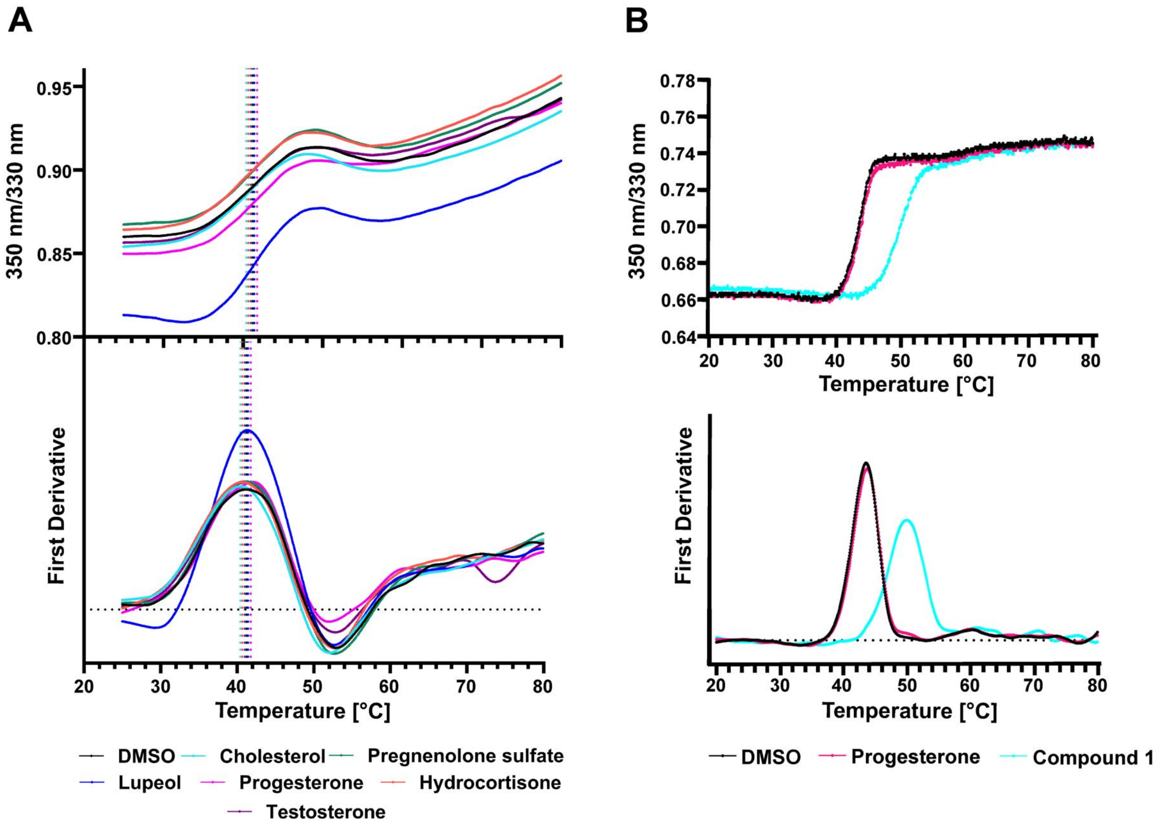

Supplementary Figure S5. Binding of steroids to ABHD2. (**A**) The binding of steroids to ABHD2$^{L33\text{-}E425}$ was assessed using differential scanning fluorimetry (DSF) following 30 min pre-incubation with 40 μM of the indicated steroids. Dashed lines denote the middle of each melting curve in the fluorescence plot, which corresponds to the peak of the first derivative curve. These lines mark the melting temperature of each sample on the X-axis. All samples showed the same melting temperature, indicating that no binding was observed in the presence of progesterone, cholesterol, testosterone, pregnenolone sulfate, hydrocortisone, lupeol, or 17α-hydroxyprogesterone. (**B**) ABHD2^{FL} melting curve assessment in the presence of 20 μM progesterone or compound **1.**
