# Supplementary material for: ABHD2 activity is not required for the non-genomic action of progesterone on human sperm
Source: Hum Reprod. 2026 May 29;41(8):1409–19. doi: 10.1093/humrep/deag085 (PMC13429874; doi:10.1093/humrep/deag085)
Supplement: deag085_Supplementary_Figure_S6 [file deag085_supplementary_figure_s6.pdf]

Human Reproduction, 2026, 1–1
https://doi.org/10.1093/humrep/deag085
Supplementary Data

human reproduction

OXFORD

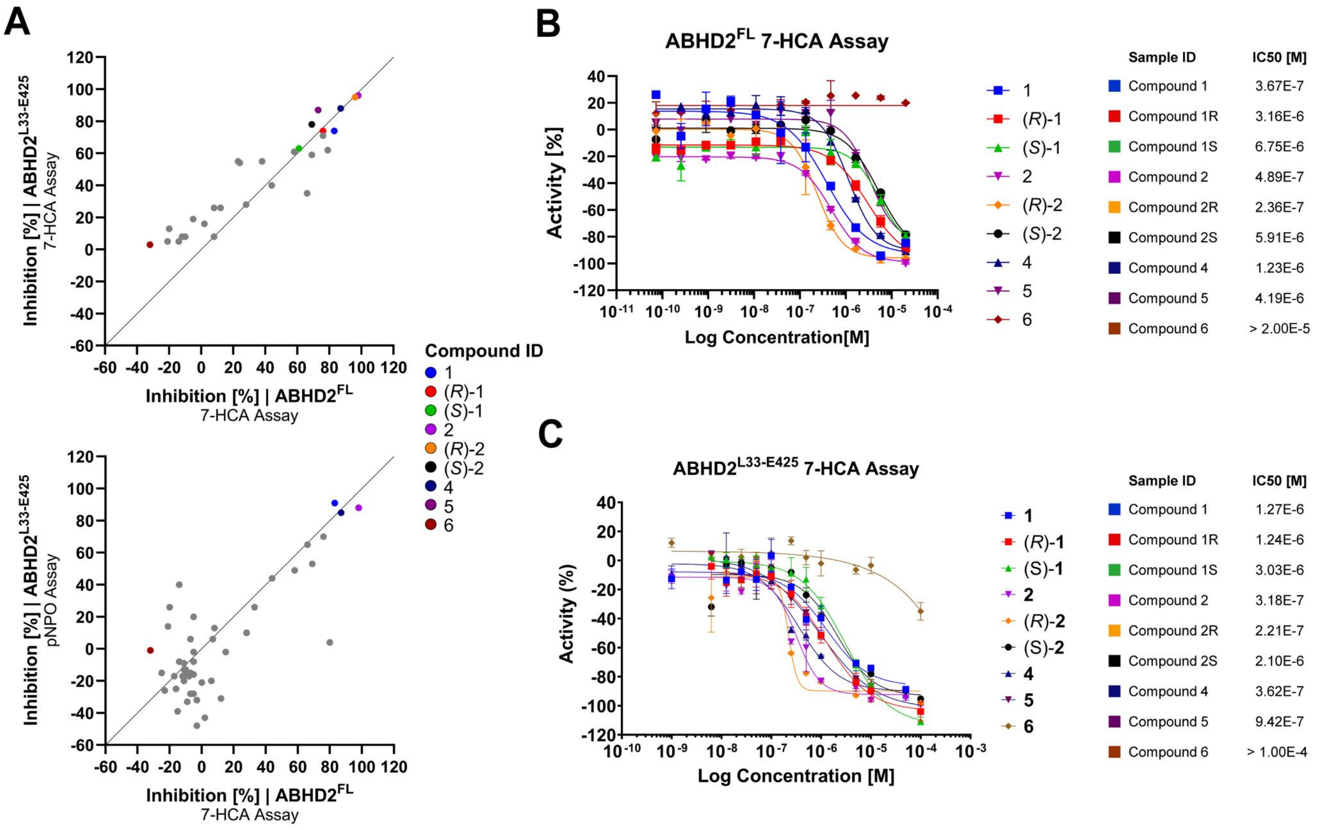

Supplementary Figure S6. Characterization of ABHD2 inhibitors. (**A**) Enzymatic activity of $ABHD2^{FL}$ and $ABHD2^{L33\text{-}E425}$ was assessed in the presence of Compound **1** derivatives. Percent inhibition of $ABHD2^{FL}$ in the 7-HCA assay at 20 μM compound concentration was correlated with percent inhibition of $ABHD2^{L33\text{-}E425}$ in the 7-HCA assay at 10 μM compound concentration (top panel) or in the pNP octanoate assay at 10 μM compound concentration (bottom panel). (**B, C**) Dose–response curves were generated by incubating a fixed $ABHD2^{FL}$ (B) or $ABHD2^{L33\text{-}E425}$ (C) concentration with 7-HCA substrate in the presence of increasing concentrations of the indicated compounds. Percent activity was plotted against log10 inhibitor concentration and fitted by nonlinear regression to obtain IC_{50} values.
