# Supplementary material for: ABHD2 activity is not required for the non-genomic action of progesterone on human sperm
Source: Hum Reprod. 2026 May 29;41(8):1409–19. doi: 10.1093/humrep/deag085 (PMC13429874; doi:10.1093/humrep/deag085)
Supplement: deag085_Supplementary_Figure_S7 [file deag085_supplementary_figure_s7.pdf]

Human Reproduction, 2026, 1–1
https://doi.org/10.1093/humrep/deag085
Supplementary Data

human reproduction
OXFORD

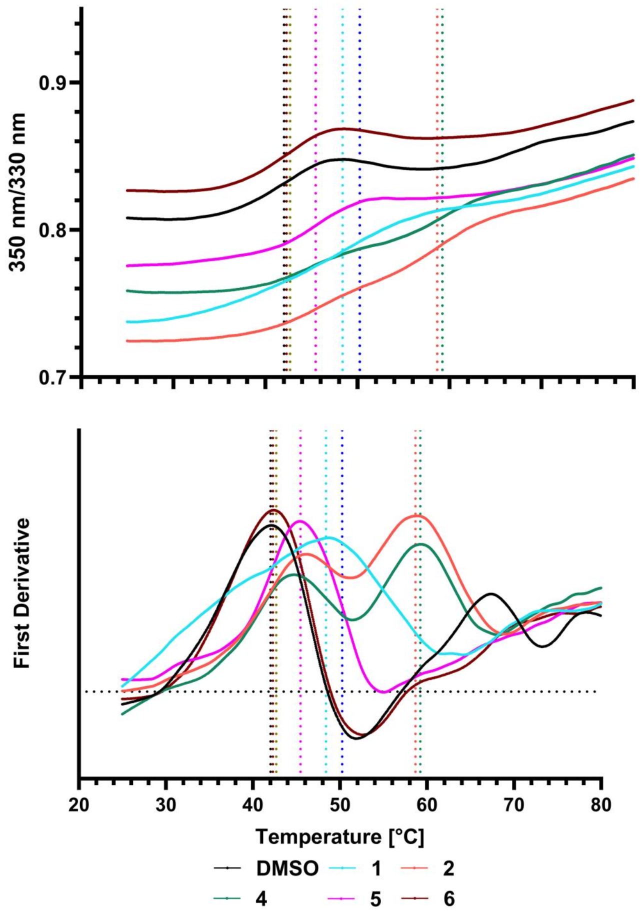

Supplementary Figure S7. Binding of small molecule compounds to ABHD2$^{L33-E425}$. The binding of small molecule compounds to ABHD2$^{L33-E425}$ was assessed using differential scanning fluorimetry (DSF) following 30 min pre-incubation with 10 μM of the indicated compounds. Dashed lines denote the middle of the melting curve in the fluorescence plot, which corresponds to the peak of the first derivative curve. These lines mark the melting temperature of each sample on the X-axis. An increased melting temperature was observed for compounds **1**, **2**, **4**, and **5**, while no stabilization was observed in the presence of compound **6.**
