# Supplementary material for: ABHD2 activity is not required for the non-genomic action of progesterone on human sperm
Source: Hum Reprod. 2026 May 29;41(8):1409–19. doi: 10.1093/humrep/deag085 (PMC13429874; doi:10.1093/humrep/deag085)
Supplement: deag085_Supplementary_Figure_S8 [file deag085_supplementary_figure_s8.pdf]

Human Reproduction, 2026, 1–1
https://doi.org/10.1093/humrep/deag085
Supplementary Data

human reproduction

OXFORD

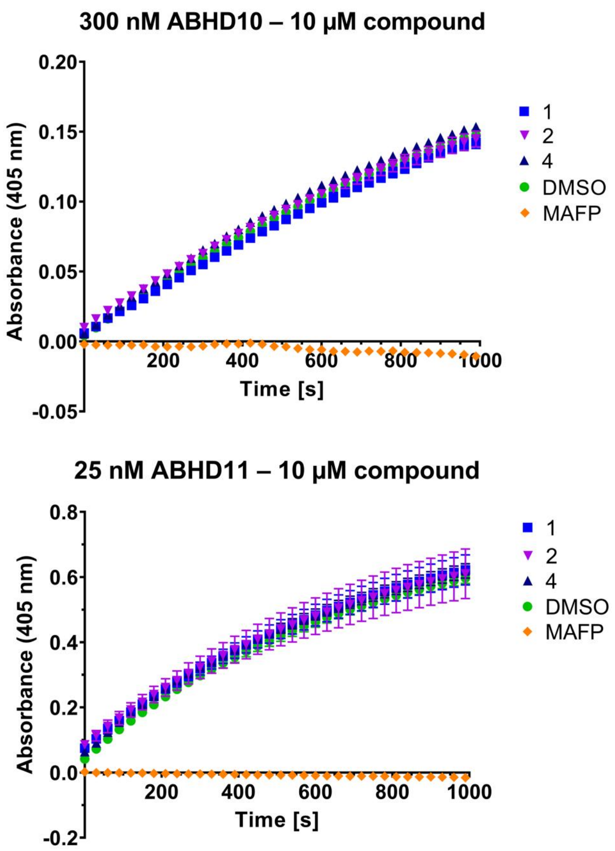

Supplementary Figure S8. ABHD2 inhibitors do not inhibit ABHD10 and ABHD11. ABHD10 and ABHD11 were assayed in a colorimetric assay using *p*-nitrophenyl butyrate as substrate. 25 nM ABHD11 and 300 nM ABHD10 were incubated for 30 min at 4°C in the presence of 10 µM compound. Concentrations were 25 nM ABHD11, 300 nM ABHD10, and 500 µM *p*-nitrophenyl butyrate. Absorbance was monitored at 405 nm continuously for 20 min in 30 s intervals on a SpectraMax iD3 (MolecularDevices). The unselective ABHD inhibitor MAFP was used as a control.
