# Supplementary material for: ABHD2 activity is not required for the non-genomic action of progesterone on human sperm
Source: Hum Reprod. 2026 May 29;41(8):1409–19. doi: 10.1093/humrep/deag085 (PMC13429874; doi:10.1093/humrep/deag085)
Supplement: deag085_Supplementary_Figure_S9 [file deag085_supplementary_figure_s9.pdf]

Human Reproduction, 2026, 1–1
https://doi.org/10.1093/humrep/deag085
Supplementary Data

human reproduction
OXFORD

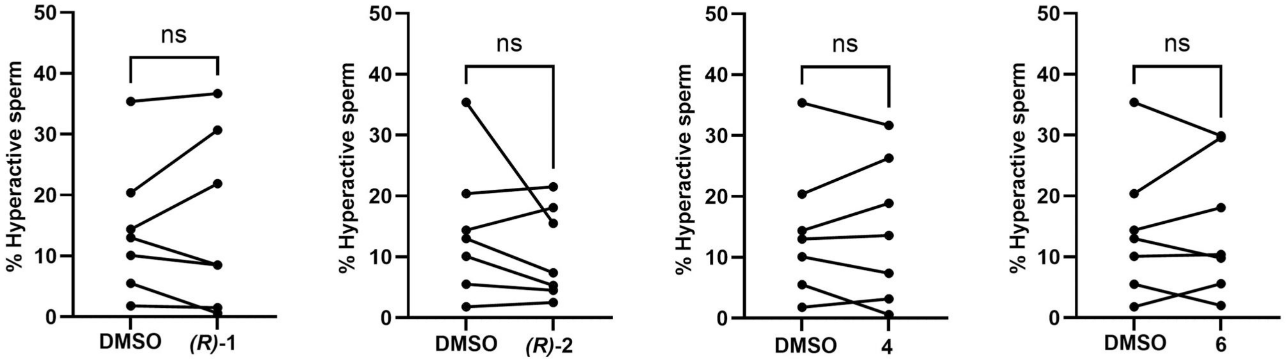

Supplementary Figure S9. ABHD2 inhibitors do not affect basal hyperactivation in human sperm. Paired plots comparing hyperactivation in the presence or absence (DMSO) of ABHD2 inhibitors. Sperm suspensions were treated with either DMSO (vehicle) or 10 μM test compound ((***R***)**-1**, (***R***)**-2**, **4**, and **6**). For each of seven independent experiments, the mean percentage of hyperactive sperm (normalized to motile sperm) across two technical replicates is shown. No statistically significant differences were detected for any comparison (not significant—NS; $P > 0.05$).
