# Supplementary material for: ABHD2 activity is not required for the non-genomic action of progesterone on human sperm
Source: Hum Reprod. 2026 May 29;41(8):1409–19. doi: 10.1093/humrep/deag085 (PMC13429874; doi:10.1093/humrep/deag085)
Supplement: deag085_Supplementary_Table_S1 [file deag085_supplementary_table_s1.pdf]

Human Reproduction, 2026, 1–1
https://doi.org/10.1093/humrep/deag085
Supplementary Data

human reproduction
OXFORD

Supplementary Table S1. Amino Acid sequence of ABHD2^{FL} construct.

1	MNAMLETPEL	PAVFDGVKLA	AVAAVLYVIV	RCLNLKSPTA	PPDLYFQDSG
51	LSRFLLKSCP	LLTKEYIPPL	IWGKSGHIQT	ALYGKMGRVR	SPHPYGHRKF
101	ITMSDGATST	FDLFEPLAEH	CVGDDITMVI	CPGIANHSEK	QYIRTFVDYA
151	QKNGYRCAVL	NHLGALPNIE	LTSPRMFTYG	CTWEFGAMVN	YIKKTYPLTQ
201	LVVVGFSLGG	NIVCKYLGET	QANQEKVLCC	VSVCQGYSAL	RAQETFMQWD
251	QCRRFYNFLM	ADNMKKIILS	HRQALFGDHV	KKPQSLEDTD	LSRLYTATSL
301	MQIDDNVMRK	FHGYNSLKEY	YEEESCMRYL	HRIYVPLMLV	NAADDPLVHE
351	SLLTIPKSLS	EKRENVMFVL	PLHGGHLGFF	EGSVLFPEPL	TWMDKLVVEY
401	ANAICQWERN	KLQCSDTEQV	EADLEENLYF	QGDPAFLYKV	VGSAAGSGEF
451	KGEELFTGVV	PILVELDGDV	NGHKFSVSGE	GEGDATYGKL	TLKFICTTGK
501	LPVPWPTLVT	TLTYGVQCFS	RYPDHMKRHD	FFKSAMPEGY	VQERTISFKD
551	DGNYKTRAEV	KFEGDTLVNR	IELKGIDFKE	DGNILGHKLE	YNYNSHNVYI
601	TADKQKNGIK	ANFKIRHNIE	DGSVQLADHY	QQNTPIGDGP	VLLPDNHYLS
651	TQSALSKDPN	EKRDHMVLLE	FVTAAGITHG	MDELYKAHHH	HHHHH

Amino acids identified in the peptides of the mass spectrometry analysis from the SDS-PAGE bands from Fig. 1 are highlighted in red.
