# Supplementary material for: ABHD2 activity is not required for the non-genomic action of progesterone on human sperm
Source: Hum Reprod. 2026 May 29;41(8):1409–19. doi: 10.1093/humrep/deag085 (PMC13429874; doi:10.1093/humrep/deag085)
Supplement: deag085_Supplementary_Table_S2 [file deag085_supplementary_table_s2.pdf]

Human Reproduction, 2026, 1–2
https://doi.org/10.1093/humrep/deag085
Supplementary Data

human reproduction OXFORD

Supplementary Table S2. IC_{50} values measured in $ABHD2^{FL}$ in the 7-HCA Assay.

Compound ID	Batch no.	# in paper	IC50 mode	IC50 [M]
CBK600192	ED0321018	1	=	3.7E-07
CBK637750	ED0321016	1R	=	3.2E-06
CBK637751	ED0321017	1S	=	6.7E-06
CBK600191	ED0321002	2	=	4.9E-07
CBK618580	ED0321003	2R	=	2.4E-07
CBK618581	ED0321004	2S	=	5.9E-06
CBK600209	DT9003016	4	=	1.2E-06
CBK618586	ED0321009	5	=	4.2E-06
CBK600171	ED0321012	6	>	2.0E-05
CBK618587	ED0321011	7	>	2.0E-05
CBK600170	DO6598002		=	1.5E-05
CBK600172	DO6598004		=	4.4E-06
CBK600173	DO6598005		>	2.0E-05
CBK600174	DO6598006		>	2.0E-05
CBK600175	DO6598007		>	2.0E-05
CBK600176	DO6598008		=	8.5E-06
CBK600177	DO6598009		>	2.0E-05
CBK600178	DO6598010		>	2.0E-05
CBK600179	DO6598011		>	2.0E-05
CBK600180	DO6598012		>	2.0E-05
CBK600181	DO6598013		>	2.0E-05
CBK600182	DO6598014		>	2.0E-05
CBK600183	DO6598015		>	2.0E-05
CBK600184	DO6598016		=	4.1E-06
CBK600185	DO6598017		>	2.0E-05
CBK600186	DO6598018		>	2.0E-05
CBK600187	DO6598019		>	2.0E-05
CBK600188	DO6598020		>	2.0E-05
CBK600189	DO6598021		>	2.0E-05
CBK600190	DO6598022		>	2.0E-05
CBK600194	DT9003001		>	2.0E-05
CBK600195	DT9003002		>	2.0E-05
CBK600196	DT9003003		=	6.4E-06
CBK600197	DT9003004		>	2.0E-05
CBK600198	DT9003005		>	2.0E-05
CBK600199	DT9003006		>	2.0E-05
CBK600200	DT9003007		>	2.0E-05
CBK600201	DT9003008		>	2.0E-05
CBK600202	DT9003009		>	2.0E-05
CBK600203	DT9003010		>	2.0E-05
CBK600204	DT9003011		>	2.0E-05
CBK600205	DT9003012		>	2.0E-05
CBK600206	DT9003013		>	2.0E-05
CBK600207	DT9003014		>	2.0E-05
CBK600208	DT9003015		=	1.1E-05
CBK600210	DT9003017		>	2.0E-05
CBK600211	DT9003018		>	2.0E-05
CBK600212	DT9003019		>	2.0E-05
CBK600213	DT9003020		>	2.0E-05
CBK600214	DT9003021		>	2.0E-05
CBK600215	DT9003022		>	2.0E-05
CBK600217	DT9004002		>	2.0E-05
CBK600219	DT9004004	HY-114157	>	2.0E-05
CBK600220	DT9004005		>	2.0E-05
CBK600221	DT9004006		>	2.0E-05
CBK600222	DT9004007		>	2.0E-05

continued

Supplementary Table S2. Continued

Compound ID	Batch no.	# in paper	IC50 mode	IC50 [M]
CBK618579	ED0321001		>	2.0E-05
CBK618582	ED0321005		>	2.0E-05
CBK618583	ED0321006		>	2.0E-05
CBK618584	ED0321007		>	2.0E-05
CBK618585	ED0321008		=	3.9E-06
CBK618588	ED0321013		>	2.0E-05
CBK618660	ED0321014		>	2.0E-05
